# Supplementary material for: Role and mechanism of NCAPD3 in promoting malignant behaviors in gastric cancer
Source: Front Pharmacol. 2024 Apr 22;15:1341039. doi: 10.3389/fphar.2024.1341039 (PMC11070777; doi:10.3389/fphar.2024.1341039)
Supplement: Supplementary file 11 [file DataSheet2.ZIP › GSEA/Transcipt factor/my_analysis.Gsea.1599461814734/gsea_report_for_NCAPD3_MUT_1599461814734.html]

Report for NCAPD3\_MUT 1599461814734 [GSEA]

| GS  follow link to MSigDB | GS DETAILS | SIZE | ES | NES | NOM p-val | FDR q-val | FWER p-val | RANK AT MAX | LEADING EDGE || 1 | RTAAACA\_FREAC2\_01 | Details ... | 88 | -0.36 | -2.71 | 0.000 | 0.003 | 0.002 | 447 | tags=63%, list=32%, signal=86% |
| 2 | TGANTCA\_AP1\_C | Details ... | 106 | -0.34 | -2.58 | 0.000 | 0.002 | 0.003 | 435 | tags=56%, list=31%, signal=74% |
| 3 | P53\_02 | Details ... | 24 | -0.53 | -2.57 | 0.000 | 0.002 | 0.004 | 229 | tags=58%, list=16%, signal=68% |
| 4 | FOXO4\_01 | Details ... | 21 | -0.52 | -2.43 | 0.000 | 0.008 | 0.024 | 279 | tags=62%, list=20%, signal=76% |
| 5 | FOXO1\_02 | Details ... | 28 | -0.47 | -2.37 | 0.000 | 0.012 | 0.044 | 436 | tags=75%, list=31%, signal=106% |
| 6 | CEBP\_C | Details ... | 23 | -0.49 | -2.31 | 0.000 | 0.017 | 0.077 | 249 | tags=48%, list=18%, signal=57% |
| 7 | GR\_01 | Details ... | 16 | -0.54 | -2.25 | 0.000 | 0.024 | 0.121 | 359 | tags=69%, list=25%, signal=91% |
| 8 | FOXO3\_01 | Details ... | 24 | -0.45 | -2.16 | 0.002 | 0.040 | 0.227 | 279 | tags=54%, list=20%, signal=66% |
| 9 | LMO2COM\_02 | Details ... | 17 | -0.51 | -2.14 | 0.000 | 0.041 | 0.252 | 403 | tags=76%, list=29%, signal=106% |
| 10 | FOXO1\_01 | Details ... | 21 | -0.46 | -2.13 | 0.000 | 0.040 | 0.268 | 293 | tags=57%, list=21%, signal=71% |
| 11 | FOXO4\_02 | Details ... | 31 | -0.39 | -2.12 | 0.000 | 0.041 | 0.301 | 455 | tags=68%, list=32%, signal=98% |
| 12 | FREAC2\_01 | Details ... | 28 | -0.42 | -2.11 | 0.005 | 0.041 | 0.319 | 293 | tags=57%, list=21%, signal=71% |
| 13 | ATF1\_Q6 | Details ... | 22 | -0.43 | -2.07 | 0.000 | 0.049 | 0.398 | 467 | tags=73%, list=33%, signal=107% |
| 14 | FREAC7\_01 | Details ... | 15 | -0.50 | -2.05 | 0.002 | 0.054 | 0.448 | 359 | tags=67%, list=25%, signal=88% |
| 15 | HFH4\_01 | Details ... | 19 | -0.45 | -2.02 | 0.005 | 0.065 | 0.531 | 276 | tags=47%, list=20%, signal=58% |
| 16 | BACH2\_01 | Details ... | 28 | -0.38 | -2.00 | 0.007 | 0.071 | 0.586 | 528 | tags=79%, list=37%, signal=123% |
| 17 | AREB6\_03 | Details ... | 21 | -0.43 | -2.00 | 0.002 | 0.066 | 0.586 | 526 | tags=81%, list=37%, signal=127% |
| 18 | HES2\_TARGET\_GENES | Details ... | 127 | -0.24 | -1.99 | 0.000 | 0.067 | 0.611 | 503 | tags=57%, list=36%, signal=80% |
| 19 | EVI1\_04 | Details ... | 18 | -0.45 | -1.97 | 0.002 | 0.075 | 0.670 | 341 | tags=61%, list=24%, signal=80% |
| 20 | CEBP\_Q3 | Details ... | 19 | -0.43 | -1.95 | 0.003 | 0.081 | 0.717 | 322 | tags=58%, list=23%, signal=74% |
| 21 | AP1\_C |  | 24 | -0.41 | -1.94 | 0.000 | 0.078 | 0.724 | 340 | tags=63%, list=24%, signal=81% |
| 22 | FREAC4\_01 |  | 17 | -0.44 | -1.90 | 0.007 | 0.096 | 0.819 | 303 | tags=59%, list=21%, signal=74% |
| 23 | CEBPA\_01 |  | 25 | -0.37 | -1.90 | 0.005 | 0.092 | 0.819 | 322 | tags=48%, list=23%, signal=61% |
| 24 | AP4\_Q6\_01 |  | 15 | -0.46 | -1.90 | 0.007 | 0.090 | 0.821 | 226 | tags=47%, list=16%, signal=55% |
| 25 | AP1\_Q6 |  | 27 | -0.37 | -1.89 | 0.010 | 0.092 | 0.842 | 468 | tags=70%, list=33%, signal=103% |
| 26 | AML1\_Q6 |  | 17 | -0.43 | -1.89 | 0.007 | 0.089 | 0.843 | 326 | tags=59%, list=23%, signal=76% |
| 27 | PAX\_Q6 |  | 24 | -0.38 | -1.89 | 0.005 | 0.086 | 0.843 | 263 | tags=50%, list=19%, signal=60% |
| 28 | AREB6\_02 |  | 23 | -0.39 | -1.88 | 0.007 | 0.084 | 0.846 | 324 | tags=52%, list=23%, signal=67% |
| 29 | GTGGGTGK\_UNKNOWN |  | 28 | -0.36 | -1.88 | 0.005 | 0.083 | 0.851 | 360 | tags=54%, list=26%, signal=70% |
| 30 | TGACAGNY\_MEIS1\_01 |  | 63 | -0.28 | -1.87 | 0.012 | 0.087 | 0.879 | 272 | tags=40%, list=19%, signal=47% |
| 31 | AML1\_01 |  | 17 | -0.43 | -1.87 | 0.012 | 0.085 | 0.882 | 326 | tags=59%, list=23%, signal=76% |
| 32 | SRF\_Q6 |  | 23 | -0.38 | -1.86 | 0.010 | 0.086 | 0.894 | 428 | tags=65%, list=30%, signal=92% |
| 33 | HIF1\_Q5 |  | 25 | -0.36 | -1.82 | 0.010 | 0.105 | 0.939 | 527 | tags=72%, list=37%, signal=113% |
| 34 | OCT1\_Q5\_01 |  | 17 | -0.42 | -1.80 | 0.010 | 0.122 | 0.963 | 34 | tags=24%, list=2%, signal=24% |
| 35 | CEBPB\_02 |  | 29 | -0.34 | -1.80 | 0.008 | 0.119 | 0.963 | 281 | tags=48%, list=20%, signal=59% |
| 36 | SRF\_C |  | 18 | -0.41 | -1.78 | 0.007 | 0.127 | 0.972 | 500 | tags=78%, list=35%, signal=119% |
| 37 | BACH1\_01 |  | 25 | -0.34 | -1.77 | 0.008 | 0.132 | 0.979 | 183 | tags=36%, list=13%, signal=41% |
| 38 | TGGNNNNNNKCCAR\_UNKNOWN |  | 32 | -0.32 | -1.77 | 0.013 | 0.129 | 0.979 | 200 | tags=34%, list=14%, signal=39% |
| 39 | DNMT3A\_TARGET\_GENES |  | 53 | -0.27 | -1.75 | 0.003 | 0.138 | 0.985 | 383 | tags=43%, list=27%, signal=57% |
| 40 | TTANTCA\_UNKNOWN |  | 86 | -0.23 | -1.75 | 0.011 | 0.139 | 0.987 | 268 | tags=33%, list=19%, signal=38% |
| 41 | YTATTTTNR\_MEF2\_02 |  | 64 | -0.25 | -1.71 | 0.014 | 0.163 | 0.993 | 276 | tags=38%, list=20%, signal=45% |
| 42 | GCANCTGNY\_MYOD\_Q6 |  | 79 | -0.24 | -1.71 | 0.006 | 0.164 | 0.993 | 253 | tags=33%, list=18%, signal=38% |
| 43 | IGLV5\_37\_TARGET\_GENES |  | 47 | -0.28 | -1.71 | 0.013 | 0.163 | 0.993 | 431 | tags=55%, list=31%, signal=77% |
| 44 | FOXD3\_01 |  | 15 | -0.42 | -1.70 | 0.024 | 0.163 | 0.993 | 632 | tags=87%, list=45%, signal=155% |
| 45 | SUPT16H\_TARGET\_GENES |  | 22 | -0.35 | -1.70 | 0.023 | 0.160 | 0.993 | 608 | tags=77%, list=43%, signal=134% |
| 46 | OCT\_Q6 |  | 17 | -0.40 | -1.70 | 0.035 | 0.157 | 0.993 | 34 | tags=24%, list=2%, signal=24% |
| 47 | DR1\_Q3 |  | 20 | -0.38 | -1.70 | 0.028 | 0.157 | 0.993 | 257 | tags=50%, list=18%, signal=60% |
| 48 | CHX10\_01 |  | 15 | -0.43 | -1.70 | 0.017 | 0.155 | 0.993 | 178 | tags=33%, list=13%, signal=38% |
| 49 | ZBTB5\_TARGET\_GENES |  | 40 | -0.29 | -1.69 | 0.015 | 0.158 | 0.993 | 151 | tags=25%, list=11%, signal=27% |
| 50 | CP2\_01 |  | 22 | -0.36 | -1.69 | 0.027 | 0.158 | 0.995 | 496 | tags=68%, list=35%, signal=104% |
| 51 | RSRFC4\_Q2 |  | 15 | -0.41 | -1.68 | 0.039 | 0.156 | 0.995 | 263 | tags=53%, list=19%, signal=65% |
| 52 | CTTTAAR\_UNKNOWN |  | 80 | -0.23 | -1.68 | 0.006 | 0.154 | 0.996 | 429 | tags=46%, list=30%, signal=63% |
| 53 | AP1\_Q6\_01 |  | 25 | -0.33 | -1.68 | 0.018 | 0.152 | 0.996 | 145 | tags=32%, list=10%, signal=35% |
| 54 | DBP\_Q6 |  | 16 | -0.39 | -1.68 | 0.026 | 0.154 | 0.996 | 435 | tags=63%, list=31%, signal=89% |
| 55 | T3R\_Q6 |  | 15 | -0.41 | -1.66 | 0.051 | 0.164 | 0.997 | 845 | tags=100%, list=60%, signal=247% |
| 56 | MEIS1\_01 |  | 22 | -0.34 | -1.66 | 0.013 | 0.166 | 0.998 | 436 | tags=59%, list=31%, signal=84% |
| 57 | HNF6\_Q6 |  | 19 | -0.38 | -1.65 | 0.043 | 0.167 | 0.998 | 252 | tags=42%, list=18%, signal=51% |
| 58 | AP1\_Q4\_01 |  | 27 | -0.33 | -1.65 | 0.025 | 0.164 | 0.998 | 486 | tags=67%, list=34%, signal=100% |
| 59 | HFH8\_01 |  | 15 | -0.40 | -1.65 | 0.034 | 0.165 | 0.998 | 403 | tags=60%, list=29%, signal=83% |
| 60 | MZF1\_02 |  | 20 | -0.35 | -1.64 | 0.034 | 0.169 | 0.998 | 34 | tags=25%, list=2%, signal=25% |
| 61 | LFA1\_Q6 |  | 18 | -0.37 | -1.63 | 0.053 | 0.177 | 0.998 | 800 | tags=94%, list=57%, signal=215% |
| 62 | AR\_01 |  | 20 | -0.35 | -1.63 | 0.034 | 0.178 | 0.999 | 342 | tags=60%, list=24%, signal=78% |
| 63 | RACCACAR\_AML\_Q6 |  | 19 | -0.36 | -1.60 | 0.029 | 0.198 | 0.999 | 293 | tags=47%, list=21%, signal=59% |
| 64 | RYTTCCTG\_ETS2\_B |  | 88 | -0.21 | -1.58 | 0.027 | 0.217 | 1.000 | 400 | tags=45%, list=28%, signal=59% |
| 65 | GATA1\_02 |  | 18 | -0.36 | -1.58 | 0.041 | 0.219 | 1.000 | 452 | tags=72%, list=32%, signal=105% |
| 66 | HSF2\_01 |  | 25 | -0.32 | -1.58 | 0.066 | 0.219 | 1.000 | 276 | tags=44%, list=20%, signal=54% |
| 67 | GREB1\_TARGET\_GENES |  | 67 | -0.23 | -1.57 | 0.032 | 0.225 | 1.000 | 482 | tags=58%, list=34%, signal=84% |
| 68 | TATA\_C |  | 25 | -0.31 | -1.56 | 0.046 | 0.232 | 1.000 | 133 | tags=28%, list=9%, signal=30% |
| 69 | GR\_Q6\_01 |  | 31 | -0.29 | -1.55 | 0.050 | 0.237 | 1.000 | 331 | tags=45%, list=23%, signal=58% |
| 70 | HP1SITEFACTOR\_Q6 |  | 16 | -0.36 | -1.55 | 0.044 | 0.237 | 1.000 | 317 | tags=50%, list=22%, signal=64% |
| 71 | ATF3\_Q6 |  | 20 | -0.35 | -1.55 | 0.063 | 0.235 | 1.000 | 416 | tags=65%, list=29%, signal=91% |
| 72 | MEF2\_Q6\_01 |  | 17 | -0.36 | -1.54 | 0.051 | 0.238 | 1.000 | 235 | tags=47%, list=17%, signal=56% |
| 73 | TATAAA\_TATA\_01 |  | 100 | -0.20 | -1.54 | 0.029 | 0.234 | 1.000 | 359 | tags=38%, list=25%, signal=47% |
| 74 | TGTTTGY\_HNF3\_Q6 |  | 57 | -0.23 | -1.54 | 0.025 | 0.239 | 1.000 | 276 | tags=33%, list=20%, signal=40% |
| 75 | HEB\_Q6 |  | 18 | -0.35 | -1.53 | 0.052 | 0.240 | 1.000 | 510 | tags=67%, list=36%, signal=103% |
| 76 | AP1\_Q4 |  | 24 | -0.31 | -1.53 | 0.067 | 0.243 | 1.000 | 324 | tags=50%, list=23%, signal=64% |
| 77 | NMYC\_01 |  | 30 | -0.29 | -1.52 | 0.054 | 0.248 | 1.000 | 435 | tags=53%, list=31%, signal=75% |
| 78 | STAT\_01 |  | 26 | -0.30 | -1.51 | 0.046 | 0.255 | 1.000 | 280 | tags=46%, list=20%, signal=57% |
| 79 | TAATTA\_CHX10\_01 |  | 58 | -0.23 | -1.51 | 0.045 | 0.264 | 1.000 | 282 | tags=33%, list=20%, signal=39% |
| 80 | E4BP4\_01 |  | 25 | -0.30 | -1.50 | 0.074 | 0.261 | 1.000 | 472 | tags=60%, list=33%, signal=89% |
| 81 | GGGYGTGNY\_UNKNOWN |  | 45 | -0.25 | -1.50 | 0.042 | 0.259 | 1.000 | 502 | tags=60%, list=36%, signal=90% |
| 82 | NFKB\_C |  | 21 | -0.32 | -1.50 | 0.060 | 0.259 | 1.000 | 572 | tags=71%, list=41%, signal=118% |
| 83 | RORA2\_01 |  | 21 | -0.32 | -1.50 | 0.067 | 0.257 | 1.000 | 440 | tags=57%, list=31%, signal=82% |
| 84 | OCT1\_02 |  | 15 | -0.36 | -1.47 | 0.065 | 0.289 | 1.000 | 202 | tags=40%, list=14%, signal=46% |
| 85 | NFAT\_Q6 |  | 17 | -0.34 | -1.47 | 0.076 | 0.288 | 1.000 | 403 | tags=59%, list=29%, signal=81% |
| 86 | COUP\_DR1\_Q6 |  | 16 | -0.35 | -1.47 | 0.089 | 0.288 | 1.000 | 253 | tags=38%, list=18%, signal=45% |
| 87 | NR0B1\_TARGET\_GENES |  | 37 | -0.27 | -1.47 | 0.088 | 0.288 | 1.000 | 322 | tags=38%, list=23%, signal=48% |
| 88 | AP1\_01 |  | 26 | -0.29 | -1.46 | 0.094 | 0.300 | 1.000 | 528 | tags=73%, list=37%, signal=115% |
| 89 | CTAWWWATA\_RSRFC4\_Q2 |  | 25 | -0.29 | -1.45 | 0.077 | 0.303 | 1.000 | 84 | tags=24%, list=6%, signal=25% |
| 90 | FREAC3\_01 |  | 29 | -0.28 | -1.45 | 0.073 | 0.302 | 1.000 | 269 | tags=41%, list=19%, signal=50% |
| 91 | CHOP\_01 |  | 29 | -0.28 | -1.45 | 0.087 | 0.300 | 1.000 | 253 | tags=38%, list=18%, signal=45% |
| 92 | ZNF236\_TARGET\_GENES |  | 26 | -0.28 | -1.45 | 0.083 | 0.301 | 1.000 | 205 | tags=35%, list=15%, signal=40% |
| 93 | CCCNNGGGAR\_OLF1\_01 |  | 27 | -0.28 | -1.45 | 0.088 | 0.299 | 1.000 | 269 | tags=37%, list=19%, signal=45% |
| 94 | MYOCD\_TARGET\_GENES |  | 98 | -0.19 | -1.45 | 0.057 | 0.300 | 1.000 | 452 | tags=47%, list=32%, signal=64% |
| 95 | ZNF423\_TARGET\_GENES |  | 93 | -0.19 | -1.44 | 0.045 | 0.299 | 1.000 | 235 | tags=27%, list=17%, signal=30% |
| 96 | FOXJ2\_TARGET\_GENES |  | 50 | -0.23 | -1.44 | 0.065 | 0.303 | 1.000 | 440 | tags=48%, list=31%, signal=67% |
| 97 | CREBP1\_01 |  | 22 | -0.29 | -1.43 | 0.105 | 0.314 | 1.000 | 472 | tags=64%, list=33%, signal=94% |
| 98 | USF\_Q6\_01 |  | 29 | -0.28 | -1.43 | 0.095 | 0.313 | 1.000 | 225 | tags=31%, list=16%, signal=36% |
| 99 | GATA1\_05 |  | 28 | -0.27 | -1.43 | 0.082 | 0.311 | 1.000 | 438 | tags=54%, list=31%, signal=76% |
| 100 | PAX2\_02 |  | 18 | -0.32 | -1.43 | 0.090 | 0.312 | 1.000 | 303 | tags=50%, list=21%, signal=63% |
| 101 | PHF21A\_TARGET\_GENES |  | 30 | -0.26 | -1.42 | 0.109 | 0.316 | 1.000 | 443 | tags=57%, list=31%, signal=81% |
| 102 | IK2\_01 |  | 23 | -0.30 | -1.42 | 0.112 | 0.320 | 1.000 | 177 | tags=35%, list=13%, signal=39% |
| 103 | AAAYWAACM\_HFH4\_01 |  | 24 | -0.29 | -1.41 | 0.092 | 0.319 | 1.000 | 381 | tags=50%, list=27%, signal=67% |
| 104 | HLF\_01 |  | 28 | -0.27 | -1.41 | 0.077 | 0.317 | 1.000 | 435 | tags=57%, list=31%, signal=81% |
| 105 | FOX\_Q2 |  | 20 | -0.31 | -1.41 | 0.123 | 0.319 | 1.000 | 510 | tags=65%, list=36%, signal=100% |
| 106 | OCT1\_B |  | 21 | -0.30 | -1.41 | 0.105 | 0.319 | 1.000 | 43 | tags=19%, list=3%, signal=19% |
| 107 | IRF\_Q6 |  | 28 | -0.27 | -1.40 | 0.099 | 0.327 | 1.000 | 400 | tags=57%, list=28%, signal=78% |
| 108 | WGGAATGY\_TEF1\_Q6 |  | 45 | -0.22 | -1.39 | 0.103 | 0.334 | 1.000 | 500 | tags=58%, list=35%, signal=87% |
| 109 | AP1FJ\_Q2 |  | 19 | -0.31 | -1.39 | 0.111 | 0.333 | 1.000 | 478 | tags=68%, list=34%, signal=102% |
| 110 | OCT\_C |  | 20 | -0.31 | -1.39 | 0.111 | 0.338 | 1.000 | 168 | tags=30%, list=12%, signal=34% |
| 111 | HNF4ALPHA\_Q6 |  | 18 | -0.31 | -1.38 | 0.117 | 0.347 | 1.000 | 326 | tags=50%, list=23%, signal=64% |
| 112 | MYCMAX\_03 |  | 22 | -0.29 | -1.38 | 0.083 | 0.345 | 1.000 | 928 | tags=95%, list=66%, signal=275% |
| 113 | MYOD\_Q6\_01 |  | 28 | -0.27 | -1.38 | 0.124 | 0.342 | 1.000 | 164 | tags=29%, list=12%, signal=32% |
| 114 | AP2REP\_01 |  | 15 | -0.34 | -1.38 | 0.132 | 0.345 | 1.000 | 200 | tags=40%, list=14%, signal=46% |
| 115 | LMO2COM\_01 |  | 18 | -0.31 | -1.38 | 0.104 | 0.343 | 1.000 | 276 | tags=44%, list=20%, signal=55% |
| 116 | MAZR\_01 |  | 16 | -0.33 | -1.37 | 0.155 | 0.354 | 1.000 | 236 | tags=31%, list=17%, signal=37% |
| 117 | SRY\_01 |  | 19 | -0.31 | -1.36 | 0.128 | 0.361 | 1.000 | 381 | tags=53%, list=27%, signal=71% |
| 118 | OCT1\_05 |  | 16 | -0.33 | -1.36 | 0.119 | 0.359 | 1.000 | 598 | tags=75%, list=42%, signal=129% |
| 119 | NKX3A\_01 |  | 15 | -0.33 | -1.36 | 0.141 | 0.359 | 1.000 | 518 | tags=73%, list=37%, signal=115% |
| 120 | HNF3B\_01 |  | 21 | -0.30 | -1.36 | 0.132 | 0.359 | 1.000 | 359 | tags=52%, list=25%, signal=69% |
| 121 | RFX1\_02 |  | 19 | -0.30 | -1.35 | 0.116 | 0.367 | 1.000 | 438 | tags=58%, list=31%, signal=83% |
| 122 | AP1\_Q2 |  | 19 | -0.30 | -1.35 | 0.151 | 0.367 | 1.000 | 399 | tags=58%, list=28%, signal=80% |
| 123 | CTGCAGY\_UNKNOWN |  | 60 | -0.20 | -1.35 | 0.118 | 0.364 | 1.000 | 200 | tags=25%, list=14%, signal=28% |
| 124 | NFKB\_Q6\_01 |  | 19 | -0.29 | -1.34 | 0.143 | 0.369 | 1.000 | 572 | tags=74%, list=41%, signal=122% |
| 125 | GATA1\_04 |  | 16 | -0.31 | -1.34 | 0.151 | 0.369 | 1.000 | 252 | tags=44%, list=18%, signal=53% |
| 126 | TGCCAAR\_NF1\_Q6 |  | 67 | -0.19 | -1.34 | 0.100 | 0.369 | 1.000 | 341 | tags=40%, list=24%, signal=51% |
| 127 | NGFIC\_01 |  | 20 | -0.30 | -1.33 | 0.144 | 0.375 | 1.000 | 74 | tags=20%, list=5%, signal=21% |
| 128 | SP1\_Q2\_01 |  | 17 | -0.31 | -1.33 | 0.139 | 0.372 | 1.000 | 436 | tags=65%, list=31%, signal=93% |
| 129 | GATA6\_01 |  | 21 | -0.29 | -1.33 | 0.139 | 0.372 | 1.000 | 301 | tags=38%, list=21%, signal=48% |
| 130 | CEBP\_Q2 |  | 28 | -0.26 | -1.33 | 0.131 | 0.375 | 1.000 | 230 | tags=32%, list=16%, signal=38% |
| 131 | RSRFC4\_01 |  | 18 | -0.30 | -1.33 | 0.141 | 0.372 | 1.000 | 513 | tags=72%, list=36%, signal=112% |
| 132 | OCT1\_07 |  | 17 | -0.30 | -1.33 | 0.150 | 0.369 | 1.000 | 219 | tags=41%, list=16%, signal=48% |
| 133 | HAND1\_TARGET\_GENES |  | 39 | -0.23 | -1.33 | 0.141 | 0.367 | 1.000 | 256 | tags=31%, list=18%, signal=37% |
| 134 | PAX4\_02 |  | 16 | -0.31 | -1.32 | 0.161 | 0.373 | 1.000 | 359 | tags=56%, list=25%, signal=75% |
| 135 | MMEF2\_Q6 |  | 15 | -0.32 | -1.30 | 0.148 | 0.399 | 1.000 | 509 | tags=73%, list=36%, signal=113% |
| 136 | P53\_DECAMER\_Q2 |  | 26 | -0.26 | -1.30 | 0.151 | 0.405 | 1.000 | 527 | tags=65%, list=37%, signal=102% |
| 137 | BDP1\_TARGET\_GENES |  | 88 | -0.18 | -1.30 | 0.114 | 0.409 | 1.000 | 389 | tags=39%, list=28%, signal=50% |
| 138 | TTCYNRGAA\_STAT5B\_01 |  | 29 | -0.24 | -1.29 | 0.158 | 0.410 | 1.000 | 280 | tags=38%, list=20%, signal=46% |
| 139 | YATGNWAAT\_OCT\_C |  | 24 | -0.26 | -1.29 | 0.171 | 0.413 | 1.000 | 369 | tags=46%, list=26%, signal=61% |
| 140 | NFKB\_Q6 |  | 23 | -0.27 | -1.29 | 0.176 | 0.416 | 1.000 | 287 | tags=39%, list=20%, signal=48% |
| 141 | PHB2\_TARGET\_GENES |  | 17 | -0.30 | -1.28 | 0.172 | 0.419 | 1.000 | 58 | tags=18%, list=4%, signal=18% |
| 142 | ZNF436\_TARGET\_GENES |  | 24 | -0.26 | -1.27 | 0.182 | 0.440 | 1.000 | 368 | tags=46%, list=26%, signal=61% |
| 143 | HSF1\_01 |  | 24 | -0.26 | -1.27 | 0.190 | 0.444 | 1.000 | 276 | tags=38%, list=20%, signal=46% |
| 144 | SRF\_Q5\_01 |  | 18 | -0.29 | -1.26 | 0.184 | 0.442 | 1.000 | 428 | tags=61%, list=30%, signal=87% |
| 145 | TEF1\_Q6 |  | 19 | -0.28 | -1.26 | 0.187 | 0.453 | 1.000 | 397 | tags=58%, list=28%, signal=79% |
| 146 | PXR\_Q2 |  | 24 | -0.26 | -1.26 | 0.178 | 0.452 | 1.000 | 482 | tags=58%, list=34%, signal=87% |
| 147 | AREB6\_01 |  | 22 | -0.27 | -1.25 | 0.170 | 0.456 | 1.000 | 478 | tags=68%, list=34%, signal=102% |
| 148 | EGR1\_01 |  | 21 | -0.27 | -1.25 | 0.180 | 0.455 | 1.000 | 183 | tags=29%, list=13%, signal=32% |
| 149 | WDHD1\_TARGET\_GENES |  | 20 | -0.27 | -1.25 | 0.181 | 0.453 | 1.000 | 209 | tags=30%, list=15%, signal=35% |
| 150 | STAT6\_01 |  | 19 | -0.28 | -1.25 | 0.197 | 0.452 | 1.000 | 248 | tags=37%, list=18%, signal=44% |
| 151 | TGANNYRGCA\_TCF11MAFG\_01 |  | 24 | -0.25 | -1.25 | 0.181 | 0.455 | 1.000 | 323 | tags=42%, list=23%, signal=53% |
| 152 | IK1\_01 |  | 26 | -0.25 | -1.24 | 0.204 | 0.456 | 1.000 | 177 | tags=27%, list=13%, signal=30% |
| 153 | STAT5B\_01 |  | 24 | -0.26 | -1.24 | 0.214 | 0.457 | 1.000 | 440 | tags=58%, list=31%, signal=83% |
| 154 | CTTTGA\_LEF1\_Q2 |  | 95 | -0.17 | -1.24 | 0.193 | 0.455 | 1.000 | 298 | tags=33%, list=21%, signal=39% |
| 155 | TBX1\_TARGET\_GENES |  | 25 | -0.25 | -1.24 | 0.211 | 0.454 | 1.000 | 917 | tags=92%, list=65%, signal=258% |
| 156 | TTCYRGAA\_UNKNOWN |  | 29 | -0.23 | -1.23 | 0.168 | 0.477 | 1.000 | 356 | tags=41%, list=25%, signal=54% |
| 157 | HMGA1\_TARGET\_GENES |  | 72 | -0.17 | -1.22 | 0.198 | 0.484 | 1.000 | 492 | tags=53%, list=35%, signal=77% |
| 158 | CTTTGT\_LEF1\_Q2 |  | 173 | -0.14 | -1.22 | 0.161 | 0.493 | 1.000 | 414 | tags=39%, list=29%, signal=48% |
| 159 | TSC22D4\_TARGET\_GENES |  | 156 | -0.14 | -1.21 | 0.170 | 0.499 | 1.000 | 452 | tags=40%, list=32%, signal=53% |
| 160 | CACBINDINGPROTEIN\_Q6 |  | 16 | -0.29 | -1.21 | 0.224 | 0.499 | 1.000 | 83 | tags=25%, list=6%, signal=26% |
| 161 | CEBPGAMMA\_Q6 |  | 24 | -0.25 | -1.21 | 0.235 | 0.497 | 1.000 | 341 | tags=38%, list=24%, signal=49% |
| 162 | OLF1\_01 |  | 15 | -0.30 | -1.21 | 0.229 | 0.495 | 1.000 | 435 | tags=53%, list=31%, signal=76% |
| 163 | MEF2\_03 |  | 17 | -0.28 | -1.21 | 0.239 | 0.496 | 1.000 | 263 | tags=41%, list=19%, signal=50% |
| 164 | CCAR2\_TARGET\_GENES |  | 59 | -0.18 | -1.20 | 0.205 | 0.502 | 1.000 | 147 | tags=19%, list=10%, signal=20% |
| 165 | ETS\_Q4 |  | 20 | -0.27 | -1.20 | 0.236 | 0.500 | 1.000 | 490 | tags=65%, list=35%, signal=98% |
| 166 | TTAYRTAA\_E4BP4\_01 |  | 32 | -0.22 | -1.20 | 0.230 | 0.500 | 1.000 | 520 | tags=66%, list=37%, signal=102% |
| 167 | HNF1\_Q6 |  | 21 | -0.26 | -1.20 | 0.254 | 0.499 | 1.000 | 253 | tags=38%, list=18%, signal=46% |
| 168 | ELK1\_01 |  | 21 | -0.25 | -1.19 | 0.220 | 0.509 | 1.000 | 435 | tags=52%, list=31%, signal=75% |
| 169 | CHAF1B\_TARGET\_GENES |  | 91 | -0.16 | -1.19 | 0.206 | 0.506 | 1.000 | 236 | tags=23%, list=17%, signal=26% |
| 170 | ZBTB44\_TARGET\_GENES |  | 66 | -0.17 | -1.19 | 0.218 | 0.510 | 1.000 | 383 | tags=41%, list=27%, signal=54% |
| 171 | SKIL\_TARGET\_GENES |  | 171 | -0.14 | -1.18 | 0.177 | 0.521 | 1.000 | 339 | tags=32%, list=24%, signal=37% |
| 172 | VDR\_Q6 |  | 21 | -0.25 | -1.18 | 0.232 | 0.521 | 1.000 | 440 | tags=52%, list=31%, signal=75% |
| 173 | MYOD\_Q6 |  | 15 | -0.29 | -1.17 | 0.249 | 0.546 | 1.000 | 510 | tags=67%, list=36%, signal=103% |
| 174 | USF\_C |  | 32 | -0.22 | -1.16 | 0.253 | 0.551 | 1.000 | 435 | tags=50%, list=31%, signal=71% |
| 175 | HNF3ALPHA\_Q6 |  | 16 | -0.27 | -1.16 | 0.269 | 0.550 | 1.000 | 253 | tags=31%, list=18%, signal=38% |
| 176 | GGGTGGRR\_PAX4\_03 |  | 110 | -0.15 | -1.16 | 0.249 | 0.547 | 1.000 | 436 | tags=42%, list=31%, signal=56% |
| 177 | MYOD\_01 |  | 22 | -0.24 | -1.16 | 0.277 | 0.549 | 1.000 | 598 | tags=73%, list=42%, signal=124% |
| 178 | AP2ALPHA\_01 |  | 17 | -0.27 | -1.16 | 0.273 | 0.546 | 1.000 | 436 | tags=53%, list=31%, signal=76% |
| 179 | AP4\_01 |  | 16 | -0.28 | -1.16 | 0.267 | 0.544 | 1.000 | 532 | tags=63%, list=38%, signal=99% |
| 180 | FOXJ2\_02 |  | 20 | -0.25 | -1.16 | 0.295 | 0.546 | 1.000 | 276 | tags=40%, list=20%, signal=49% |
| 181 | ZNF654\_TARGET\_GENES |  | 133 | -0.14 | -1.16 | 0.224 | 0.544 | 1.000 | 183 | tags=18%, list=13%, signal=19% |
| 182 | USF\_Q6 |  | 29 | -0.22 | -1.16 | 0.258 | 0.543 | 1.000 | 259 | tags=31%, list=18%, signal=37% |
| 183 | SRF\_Q4 |  | 16 | -0.28 | -1.15 | 0.276 | 0.549 | 1.000 | 216 | tags=38%, list=15%, signal=44% |
| 184 | YCATTAA\_UNKNOWN |  | 40 | -0.19 | -1.15 | 0.294 | 0.546 | 1.000 | 334 | tags=38%, list=24%, signal=48% |
| 185 | HNF4\_DR1\_Q3 |  | 18 | -0.27 | -1.15 | 0.284 | 0.544 | 1.000 | 95 | tags=22%, list=7%, signal=24% |
| 186 | POU1F1\_Q6 |  | 17 | -0.26 | -1.15 | 0.291 | 0.543 | 1.000 | 667 | tags=82%, list=47%, signal=154% |
| 187 | FOXJ2\_01 |  | 17 | -0.27 | -1.14 | 0.317 | 0.550 | 1.000 | 403 | tags=53%, list=29%, signal=73% |
| 188 | CART1\_01 |  | 21 | -0.24 | -1.14 | 0.280 | 0.554 | 1.000 | 29 | tags=14%, list=2%, signal=14% |
| 189 | SOX10\_TARGET\_GENES |  | 22 | -0.24 | -1.14 | 0.283 | 0.558 | 1.000 | 315 | tags=41%, list=22%, signal=52% |
| 190 | RORA1\_01 |  | 19 | -0.26 | -1.14 | 0.303 | 0.557 | 1.000 | 50 | tags=16%, list=4%, signal=16% |
| 191 | MYCMAX\_01 |  | 28 | -0.22 | -1.13 | 0.287 | 0.558 | 1.000 | 1101 | tags=100%, list=78%, signal=446% |
| 192 | ICSBP\_Q6 |  | 26 | -0.23 | -1.13 | 0.310 | 0.557 | 1.000 | 370 | tags=50%, list=26%, signal=67% |
| 193 | SOX5\_01 |  | 22 | -0.23 | -1.13 | 0.309 | 0.563 | 1.000 | 527 | tags=64%, list=37%, signal=100% |
| 194 | OCT1\_01 |  | 19 | -0.25 | -1.13 | 0.291 | 0.564 | 1.000 | 207 | tags=32%, list=15%, signal=37% |
| 195 | TAL1BETAE47\_01 |  | 19 | -0.25 | -1.12 | 0.298 | 0.568 | 1.000 | 484 | tags=58%, list=34%, signal=87% |
| 196 | HIF1\_Q3 |  | 26 | -0.23 | -1.12 | 0.277 | 0.570 | 1.000 | 279 | tags=35%, list=20%, signal=42% |
| 197 | ZNF33A\_TARGET\_GENES |  | 21 | -0.24 | -1.12 | 0.288 | 0.569 | 1.000 | 612 | tags=71%, list=43%, signal=124% |
| 198 | NFKAPPAB65\_01 |  | 22 | -0.23 | -1.12 | 0.333 | 0.577 | 1.000 | 573 | tags=68%, list=41%, signal=113% |
| 199 | MYB\_Q5\_01 |  | 25 | -0.22 | -1.11 | 0.330 | 0.589 | 1.000 | 260 | tags=32%, list=18%, signal=39% |
| 200 | TFIIA\_Q6 |  | 19 | -0.25 | -1.11 | 0.347 | 0.587 | 1.000 | 276 | tags=42%, list=20%, signal=52% |
| 201 | TAL1ALPHAE47\_01 |  | 21 | -0.24 | -1.11 | 0.336 | 0.585 | 1.000 | 510 | tags=62%, list=36%, signal=96% |
| 202 | TAZ\_TARGET\_GENES |  | 31 | -0.21 | -1.11 | 0.323 | 0.588 | 1.000 | 393 | tags=48%, list=28%, signal=66% |
| 203 | E2F1\_Q4 |  | 33 | -0.20 | -1.10 | 0.335 | 0.595 | 1.000 | 1022 | tags=94%, list=72%, signal=333% |
| 204 | HDAC8\_TARGET\_GENES |  | 19 | -0.24 | -1.10 | 0.322 | 0.596 | 1.000 | 942 | tags=95%, list=67%, signal=281% |
| 205 | NKX22\_01 |  | 17 | -0.26 | -1.10 | 0.333 | 0.593 | 1.000 | 269 | tags=35%, list=19%, signal=43% |
| 206 | TTCNRGNNNNTTC\_HSF\_Q6 |  | 15 | -0.27 | -1.10 | 0.309 | 0.593 | 1.000 | 154 | tags=27%, list=11%, signal=30% |
| 207 | RNGTGGGC\_UNKNOWN |  | 63 | -0.16 | -1.09 | 0.346 | 0.613 | 1.000 | 95 | tags=14%, list=7%, signal=15% |
| 208 | TOX4\_TARGET\_GENES |  | 17 | -0.25 | -1.09 | 0.340 | 0.612 | 1.000 | 332 | tags=41%, list=24%, signal=53% |
| 209 | LEF1\_Q6 |  | 21 | -0.23 | -1.08 | 0.343 | 0.618 | 1.000 | 202 | tags=29%, list=14%, signal=33% |
| 210 | MAML1\_TARGET\_GENES |  | 15 | -0.26 | -1.08 | 0.350 | 0.619 | 1.000 | 626 | tags=73%, list=44%, signal=130% |
| 211 | RGAANNTTC\_HSF1\_01 |  | 40 | -0.18 | -1.08 | 0.359 | 0.623 | 1.000 | 276 | tags=30%, list=20%, signal=36% |
| 212 | SRY\_02 |  | 26 | -0.22 | -1.08 | 0.379 | 0.622 | 1.000 | 341 | tags=42%, list=24%, signal=55% |
| 213 | PPAR\_DR1\_Q2 |  | 16 | -0.26 | -1.07 | 0.362 | 0.622 | 1.000 | 159 | tags=31%, list=11%, signal=35% |
| 214 | AACTTT\_UNKNOWN |  | 162 | -0.13 | -1.07 | 0.327 | 0.623 | 1.000 | 209 | tags=21%, list=15%, signal=22% |
| 215 | EFC\_Q6 |  | 26 | -0.22 | -1.07 | 0.365 | 0.629 | 1.000 | 66 | tags=15%, list=5%, signal=16% |
| 216 | PAX5\_01 |  | 17 | -0.25 | -1.06 | 0.401 | 0.646 | 1.000 | 92 | tags=24%, list=7%, signal=25% |
| 217 | CEBP\_01 |  | 25 | -0.21 | -1.06 | 0.377 | 0.647 | 1.000 | 359 | tags=44%, list=25%, signal=58% |
| 218 | OCT1\_Q6 |  | 23 | -0.22 | -1.06 | 0.395 | 0.648 | 1.000 | 43 | tags=13%, list=3%, signal=13% |
| 219 | GATA1\_01 |  | 16 | -0.25 | -1.05 | 0.415 | 0.654 | 1.000 | 823 | tags=88%, list=58%, signal=208% |
| 220 | PEA3\_Q6 |  | 20 | -0.23 | -1.05 | 0.366 | 0.651 | 1.000 | 177 | tags=25%, list=13%, signal=28% |
| 221 | NR1H4\_TARGET\_GENES |  | 31 | -0.20 | -1.04 | 0.406 | 0.663 | 1.000 | 338 | tags=39%, list=24%, signal=50% |
| 222 | PBXIP1\_TARGET\_GENES |  | 17 | -0.24 | -1.04 | 0.397 | 0.670 | 1.000 | 591 | tags=71%, list=42%, signal=120% |
| 223 | WGTTNNNNNAAA\_UNKNOWN |  | 55 | -0.16 | -1.03 | 0.424 | 0.680 | 1.000 | 341 | tags=33%, list=24%, signal=41% |
| 224 | ATF5\_TARGET\_GENES |  | 38 | -0.18 | -1.03 | 0.437 | 0.685 | 1.000 | 43 | tags=11%, list=3%, signal=11% |
| 225 | TCF11\_01 |  | 16 | -0.24 | -1.02 | 0.434 | 0.705 | 1.000 | 1075 | tags=100%, list=76%, signal=415% |
| 226 | CGTSACG\_PAX3\_B |  | 15 | -0.25 | -1.02 | 0.431 | 0.707 | 1.000 | 1065 | tags=100%, list=75%, signal=403% |
| 227 | ZNF589\_TARGET\_GENES |  | 60 | -0.15 | -1.01 | 0.430 | 0.713 | 1.000 | 201 | tags=20%, list=14%, signal=22% |
| 228 | TEL2\_Q6 |  | 19 | -0.22 | -1.01 | 0.420 | 0.710 | 1.000 | 459 | tags=53%, list=33%, signal=77% |
| 229 | SOX6\_TARGET\_GENES |  | 171 | -0.12 | -1.01 | 0.443 | 0.713 | 1.000 | 131 | tags=12%, list=9%, signal=12% |
| 230 | PAX4\_01 |  | 27 | -0.20 | -1.01 | 0.416 | 0.713 | 1.000 | 329 | tags=41%, list=23%, signal=52% |
| 231 | NRF2\_Q4 |  | 20 | -0.23 | -1.01 | 0.447 | 0.711 | 1.000 | 303 | tags=35%, list=21%, signal=44% |
| 232 | E12\_Q6 |  | 20 | -0.22 | -1.01 | 0.433 | 0.716 | 1.000 | 500 | tags=60%, list=35%, signal=92% |
| 233 | RYBP\_TARGET\_GENES |  | 145 | -0.12 | -1.00 | 0.477 | 0.716 | 1.000 | 228 | tags=21%, list=16%, signal=23% |
| 234 | SOX9\_B1 |  | 20 | -0.22 | -1.00 | 0.452 | 0.718 | 1.000 | 158 | tags=20%, list=11%, signal=22% |
| 235 | HNF3\_Q6 |  | 19 | -0.22 | -1.00 | 0.460 | 0.716 | 1.000 | 632 | tags=74%, list=45%, signal=132% |
| 236 | NFAT\_Q4\_01 |  | 15 | -0.24 | -1.00 | 0.442 | 0.713 | 1.000 | 403 | tags=53%, list=29%, signal=74% |
| 237 | IPF1\_Q4 |  | 17 | -0.24 | -1.00 | 0.476 | 0.724 | 1.000 | 77 | tags=18%, list=5%, signal=18% |
| 238 | CREB\_Q4\_01 |  | 15 | -0.24 | -0.99 | 0.455 | 0.723 | 1.000 | 484 | tags=60%, list=34%, signal=90% |
| 239 | HNF1A\_TARGET\_GENES |  | 164 | -0.12 | -0.99 | 0.477 | 0.726 | 1.000 | 86 | tags=10%, list=6%, signal=10% |
| 240 | PSMB5\_TARGET\_GENES |  | 33 | -0.18 | -0.99 | 0.476 | 0.727 | 1.000 | 657 | tags=73%, list=47%, signal=133% |
| 241 | AFP1\_Q6 |  | 17 | -0.23 | -0.99 | 0.465 | 0.726 | 1.000 | 146 | tags=24%, list=10%, signal=26% |
| 242 | TATA\_01 |  | 19 | -0.22 | -0.99 | 0.484 | 0.726 | 1.000 | 84 | tags=16%, list=6%, signal=17% |
| 243 | TGIF\_01 |  | 19 | -0.22 | -0.99 | 0.476 | 0.725 | 1.000 | 279 | tags=37%, list=20%, signal=45% |
| 244 | YYCATTCAWW\_UNKNOWN |  | 20 | -0.21 | -0.99 | 0.449 | 0.724 | 1.000 | 391 | tags=45%, list=28%, signal=61% |
| 245 | TGASTMAGC\_NFE2\_01 |  | 21 | -0.21 | -0.98 | 0.479 | 0.725 | 1.000 | 528 | tags=62%, list=37%, signal=97% |
| 246 | CAGGTA\_AREB6\_01 |  | 59 | -0.15 | -0.98 | 0.487 | 0.737 | 1.000 | 252 | tags=27%, list=18%, signal=32% |
| 247 | HDAC3\_TARGET\_GENES |  | 189 | -0.11 | -0.97 | 0.528 | 0.739 | 1.000 | 215 | tags=19%, list=15%, signal=19% |
| 248 | GATA1\_03 |  | 20 | -0.21 | -0.97 | 0.499 | 0.738 | 1.000 | 484 | tags=55%, list=34%, signal=83% |
| 249 | ZNF92\_TARGET\_GENES |  | 98 | -0.13 | -0.97 | 0.531 | 0.742 | 1.000 | 935 | tags=85%, list=66%, signal=234% |
| 250 | ELF1\_Q6 |  | 21 | -0.21 | -0.96 | 0.493 | 0.751 | 1.000 | 59 | tags=14%, list=4%, signal=15% |
| 251 | F10\_TARGET\_GENES |  | 17 | -0.22 | -0.96 | 0.491 | 0.755 | 1.000 | 184 | tags=29%, list=13%, signal=33% |
| 252 | CEBPB\_01 |  | 27 | -0.19 | -0.96 | 0.494 | 0.754 | 1.000 | 306 | tags=33%, list=22%, signal=42% |
| 253 | ER\_Q6\_01 |  | 24 | -0.19 | -0.96 | 0.494 | 0.759 | 1.000 | 334 | tags=38%, list=24%, signal=48% |
| 254 | OSF2\_Q6 |  | 18 | -0.22 | -0.96 | 0.496 | 0.760 | 1.000 | 302 | tags=39%, list=21%, signal=49% |
| 255 | MAFG\_TARGET\_GENES |  | 148 | -0.11 | -0.95 | 0.564 | 0.771 | 1.000 | 535 | tags=49%, list=38%, signal=70% |
| 256 | AP2\_Q6\_01 |  | 18 | -0.21 | -0.94 | 0.527 | 0.777 | 1.000 | 276 | tags=39%, list=20%, signal=48% |
| 257 | AMEF2\_Q6 |  | 23 | -0.19 | -0.94 | 0.556 | 0.779 | 1.000 | 510 | tags=57%, list=36%, signal=87% |
| 258 | TST1\_01 |  | 18 | -0.21 | -0.94 | 0.511 | 0.780 | 1.000 | 943 | tags=94%, list=67%, signal=281% |
| 259 | NF1\_Q6\_01 |  | 18 | -0.22 | -0.94 | 0.496 | 0.780 | 1.000 | 195 | tags=28%, list=14%, signal=32% |
| 260 | ETS2\_B |  | 19 | -0.21 | -0.94 | 0.540 | 0.783 | 1.000 | 459 | tags=58%, list=33%, signal=85% |
| 261 | NFKAPPAB\_01 |  | 25 | -0.19 | -0.94 | 0.556 | 0.782 | 1.000 | 573 | tags=64%, list=41%, signal=106% |
| 262 | ZNF175\_TARGET\_GENES |  | 82 | -0.13 | -0.93 | 0.612 | 0.798 | 1.000 | 120 | tags=12%, list=9%, signal=13% |
| 263 | UBN1\_TARGET\_GENES |  | 163 | -0.11 | -0.92 | 0.606 | 0.807 | 1.000 | 360 | tags=31%, list=26%, signal=37% |
| 264 | NKX61\_01 |  | 22 | -0.20 | -0.92 | 0.551 | 0.807 | 1.000 | 199 | tags=27%, list=14%, signal=31% |
| 265 | GGATTA\_PITX2\_Q2 |  | 45 | -0.15 | -0.92 | 0.569 | 0.811 | 1.000 | 374 | tags=38%, list=27%, signal=50% |
| 266 | ZNF584\_TARGET\_GENES |  | 42 | -0.15 | -0.91 | 0.580 | 0.818 | 1.000 | 964 | tags=88%, list=68%, signal=270% |
| 267 | HOXA4\_Q2 |  | 27 | -0.18 | -0.91 | 0.567 | 0.818 | 1.000 | 74 | tags=15%, list=5%, signal=15% |
| 268 | NCOA6\_TARGET\_GENES |  | 35 | -0.16 | -0.91 | 0.598 | 0.822 | 1.000 | 276 | tags=31%, list=20%, signal=38% |
| 269 | TCF1P\_Q6 |  | 20 | -0.20 | -0.90 | 0.593 | 0.833 | 1.000 | 359 | tags=40%, list=25%, signal=53% |
| 270 | HNF4\_Q6 |  | 28 | -0.17 | -0.89 | 0.614 | 0.840 | 1.000 | 363 | tags=39%, list=26%, signal=52% |
| 271 | HBZ\_TARGET\_GENES |  | 89 | -0.12 | -0.89 | 0.646 | 0.837 | 1.000 | 253 | tags=22%, list=18%, signal=26% |
| 272 | TBP\_01 |  | 22 | -0.19 | -0.89 | 0.597 | 0.843 | 1.000 | 415 | tags=45%, list=29%, signal=63% |
| 273 | ZSCAN4\_TARGET\_GENES |  | 92 | -0.12 | -0.89 | 0.637 | 0.842 | 1.000 | 207 | tags=20%, list=15%, signal=21% |
| 274 | P300\_01 |  | 21 | -0.19 | -0.89 | 0.581 | 0.843 | 1.000 | 297 | tags=38%, list=21%, signal=48% |
| 275 | AEBP2\_TARGET\_GENES |  | 109 | -0.11 | -0.88 | 0.683 | 0.851 | 1.000 | 336 | tags=29%, list=24%, signal=36% |
| 276 | PRDM4\_TARGET\_GENES |  | 41 | -0.15 | -0.88 | 0.615 | 0.848 | 1.000 | 332 | tags=32%, list=24%, signal=40% |
| 277 | BRN2\_01 |  | 22 | -0.19 | -0.88 | 0.607 | 0.848 | 1.000 | 403 | tags=45%, list=29%, signal=63% |
| 278 | HNF1\_01 |  | 18 | -0.20 | -0.88 | 0.625 | 0.847 | 1.000 | 218 | tags=28%, list=15%, signal=32% |
| 279 | POU6F1\_01 |  | 17 | -0.21 | -0.88 | 0.617 | 0.847 | 1.000 | 257 | tags=35%, list=18%, signal=43% |
| 280 | ARP1\_01 |  | 16 | -0.20 | -0.87 | 0.607 | 0.854 | 1.000 | 383 | tags=44%, list=27%, signal=59% |
| 281 | NKX62\_Q2 |  | 16 | -0.21 | -0.87 | 0.644 | 0.856 | 1.000 | 253 | tags=31%, list=18%, signal=38% |
| 282 | ZIC3\_01 |  | 19 | -0.19 | -0.87 | 0.636 | 0.853 | 1.000 | 92 | tags=16%, list=7%, signal=17% |
| 283 | SMAD\_Q6 |  | 23 | -0.17 | -0.87 | 0.629 | 0.854 | 1.000 | 66 | tags=13%, list=5%, signal=13% |
| 284 | GR\_Q6 |  | 17 | -0.20 | -0.86 | 0.630 | 0.856 | 1.000 | 1132 | tags=100%, list=80%, signal=500% |
| 285 | ZNF350\_TARGET\_GENES |  | 117 | -0.11 | -0.86 | 0.721 | 0.856 | 1.000 | 260 | tags=23%, list=18%, signal=26% |
| 286 | BPTF\_TARGET\_GENES |  | 100 | -0.11 | -0.86 | 0.709 | 0.863 | 1.000 | 452 | tags=41%, list=32%, signal=56% |
| 287 | CREB3L4\_TARGET\_GENES |  | 144 | -0.10 | -0.86 | 0.744 | 0.860 | 1.000 | 335 | tags=28%, list=24%, signal=33% |
| 288 | ZNF597\_TARGET\_GENES |  | 64 | -0.13 | -0.86 | 0.651 | 0.861 | 1.000 | 249 | tags=25%, list=18%, signal=29% |
| 289 | CDPCR3HD\_01 |  | 22 | -0.18 | -0.85 | 0.631 | 0.865 | 1.000 | 108 | tags=18%, list=8%, signal=19% |
| 290 | ZNF10\_TARGET\_GENES |  | 73 | -0.12 | -0.85 | 0.707 | 0.863 | 1.000 | 236 | tags=22%, list=17%, signal=25% |
| 291 | ZNF547\_TARGET\_GENES |  | 36 | -0.15 | -0.85 | 0.684 | 0.868 | 1.000 | 263 | tags=28%, list=19%, signal=33% |
| 292 | CEBPDELTA\_Q6 |  | 23 | -0.17 | -0.85 | 0.642 | 0.866 | 1.000 | 207 | tags=26%, list=15%, signal=30% |
| 293 | MEF2D\_TARGET\_GENES |  | 53 | -0.13 | -0.83 | 0.692 | 0.899 | 1.000 | 120 | tags=13%, list=9%, signal=14% |
| 294 | CREL\_01 |  | 25 | -0.16 | -0.82 | 0.700 | 0.899 | 1.000 | 573 | tags=64%, list=41%, signal=106% |
| 295 | EMX1\_TARGET\_GENES |  | 22 | -0.17 | -0.82 | 0.711 | 0.901 | 1.000 | 276 | tags=32%, list=20%, signal=39% |
| 296 | E2A\_Q2 |  | 23 | -0.17 | -0.82 | 0.682 | 0.899 | 1.000 | 532 | tags=57%, list=38%, signal=89% |
| 297 | STAT\_Q6 |  | 29 | -0.16 | -0.82 | 0.693 | 0.898 | 1.000 | 276 | tags=28%, list=20%, signal=34% |
| 298 | USF\_02 |  | 34 | -0.14 | -0.82 | 0.700 | 0.900 | 1.000 | 159 | tags=18%, list=11%, signal=19% |
| 299 | ZF5\_01 |  | 20 | -0.18 | -0.81 | 0.749 | 0.902 | 1.000 | 1047 | tags=95%, list=74%, signal=363% |
| 300 | MYB\_Q3 |  | 20 | -0.17 | -0.81 | 0.681 | 0.904 | 1.000 | 154 | tags=20%, list=11%, signal=22% |
| 301 | SFMBT1\_TARGET\_GENES |  | 166 | -0.09 | -0.80 | 0.804 | 0.913 | 1.000 | 350 | tags=30%, list=25%, signal=35% |
| 302 | KLF3\_TARGET\_GENES |  | 80 | -0.11 | -0.80 | 0.769 | 0.913 | 1.000 | 472 | tags=43%, list=33%, signal=60% |
| 303 | DACH1\_TARGET\_GENES |  | 76 | -0.11 | -0.80 | 0.758 | 0.910 | 1.000 | 220 | tags=20%, list=16%, signal=22% |
| 304 | TGAYRTCA\_ATF3\_Q6 |  | 50 | -0.13 | -0.80 | 0.732 | 0.908 | 1.000 | 393 | tags=36%, list=28%, signal=48% |
| 305 | GATTGGY\_NFY\_Q6\_01 |  | 112 | -0.10 | -0.79 | 0.809 | 0.928 | 1.000 | 419 | tags=37%, list=30%, signal=48% |
| 306 | OCT1\_04 |  | 19 | -0.18 | -0.79 | 0.708 | 0.926 | 1.000 | 207 | tags=26%, list=15%, signal=30% |
| 307 | USF\_01 |  | 31 | -0.15 | -0.79 | 0.733 | 0.924 | 1.000 | 272 | tags=26%, list=19%, signal=31% |
| 308 | SMAD4\_Q6 |  | 21 | -0.17 | -0.78 | 0.755 | 0.927 | 1.000 | 72 | tags=14%, list=5%, signal=15% |
| 309 | WRNIP1\_TARGET\_GENES |  | 114 | -0.10 | -0.78 | 0.861 | 0.929 | 1.000 | 155 | tags=13%, list=11%, signal=14% |
| 310 | AP2\_Q6 |  | 17 | -0.18 | -0.78 | 0.766 | 0.930 | 1.000 | 200 | tags=24%, list=14%, signal=27% |
| 311 | EGR2\_01 |  | 19 | -0.18 | -0.78 | 0.754 | 0.927 | 1.000 | 471 | tags=53%, list=33%, signal=78% |
| 312 | NFY\_Q6 |  | 22 | -0.16 | -0.77 | 0.732 | 0.931 | 1.000 | 130 | tags=18%, list=9%, signal=20% |
| 313 | ADNP\_TARGET\_GENES |  | 49 | -0.13 | -0.77 | 0.806 | 0.931 | 1.000 | 537 | tags=51%, list=38%, signal=80% |
| 314 | ARNT\_01 |  | 38 | -0.13 | -0.77 | 0.786 | 0.930 | 1.000 | 189 | tags=18%, list=13%, signal=21% |
| 315 | AHR\_Q5 |  | 21 | -0.17 | -0.77 | 0.783 | 0.928 | 1.000 | 119 | tags=19%, list=8%, signal=20% |
| 316 | DR4\_Q2 |  | 29 | -0.14 | -0.77 | 0.749 | 0.926 | 1.000 | 317 | tags=31%, list=22%, signal=39% |
| 317 | ZNF394\_TARGET\_GENES |  | 101 | -0.10 | -0.77 | 0.859 | 0.924 | 1.000 | 230 | tags=20%, list=16%, signal=22% |
| 318 | CREB3\_TARGET\_GENES |  | 34 | -0.14 | -0.77 | 0.777 | 0.924 | 1.000 | 1054 | tags=94%, list=75%, signal=363% |
| 319 | MYCMAX\_02 |  | 25 | -0.15 | -0.76 | 0.781 | 0.923 | 1.000 | 137 | tags=16%, list=10%, signal=17% |
| 320 | CREB\_Q2 |  | 25 | -0.16 | -0.76 | 0.759 | 0.928 | 1.000 | 308 | tags=36%, list=22%, signal=45% |
| 321 | ZBTB12\_TARGET\_GENES |  | 126 | -0.09 | -0.76 | 0.880 | 0.927 | 1.000 | 96 | tags=10%, list=7%, signal=9% |
| 322 | LHX9\_TARGET\_GENES |  | 89 | -0.10 | -0.76 | 0.842 | 0.925 | 1.000 | 429 | tags=38%, list=30%, signal=51% |
| 323 | LBP1\_Q6 |  | 16 | -0.18 | -0.75 | 0.767 | 0.925 | 1.000 | 35 | tags=13%, list=2%, signal=13% |
| 324 | NCOA4\_TARGET\_GENES |  | 85 | -0.10 | -0.75 | 0.845 | 0.924 | 1.000 | 121 | tags=12%, list=9%, signal=12% |
| 325 | NF1\_Q6 |  | 21 | -0.16 | -0.75 | 0.795 | 0.926 | 1.000 | 543 | tags=57%, list=38%, signal=92% |
| 326 | TASOR\_TARGET\_GENES |  | 27 | -0.15 | -0.74 | 0.781 | 0.933 | 1.000 | 844 | tags=81%, list=60%, signal=199% |
| 327 | TGACATY\_UNKNOWN |  | 57 | -0.11 | -0.74 | 0.854 | 0.937 | 1.000 | 264 | tags=25%, list=19%, signal=29% |
| 328 | EPC1\_TARGET\_GENES |  | 42 | -0.12 | -0.74 | 0.813 | 0.936 | 1.000 | 1115 | tags=95%, list=79%, signal=440% |
| 329 | TGACGTCA\_ATF3\_Q6 |  | 20 | -0.16 | -0.74 | 0.794 | 0.935 | 1.000 | 373 | tags=40%, list=26%, signal=54% |
| 330 | MAF\_Q6 |  | 20 | -0.16 | -0.73 | 0.807 | 0.939 | 1.000 | 30 | tags=10%, list=2%, signal=10% |
| 331 | STAT3\_02 |  | 15 | -0.17 | -0.73 | 0.816 | 0.940 | 1.000 | 140 | tags=20%, list=10%, signal=22% |
| 332 | ZNF660\_TARGET\_GENES |  | 70 | -0.11 | -0.73 | 0.869 | 0.940 | 1.000 | 344 | tags=30%, list=24%, signal=38% |
| 333 | ZNF664\_TARGET\_GENES |  | 70 | -0.10 | -0.72 | 0.851 | 0.937 | 1.000 | 469 | tags=40%, list=33%, signal=57% |
| 334 | TCF7\_TARGET\_GENES |  | 186 | -0.08 | -0.72 | 0.934 | 0.935 | 1.000 | 503 | tags=42%, list=36%, signal=57% |
| 335 | ZNF512\_TARGET\_GENES |  | 31 | -0.13 | -0.72 | 0.818 | 0.933 | 1.000 | 82 | tags=13%, list=6%, signal=13% |
| 336 | OCT1\_03 |  | 18 | -0.16 | -0.72 | 0.848 | 0.938 | 1.000 | 435 | tags=44%, list=31%, signal=63% |
| 337 | CDC73\_TARGET\_GENES |  | 31 | -0.13 | -0.72 | 0.814 | 0.937 | 1.000 | 990 | tags=90%, list=70%, signal=296% |
| 338 | DNMT1\_TARGET\_GENES |  | 17 | -0.17 | -0.72 | 0.815 | 0.935 | 1.000 | 183 | tags=24%, list=13%, signal=27% |
| 339 | CRX\_Q4 |  | 17 | -0.16 | -0.71 | 0.811 | 0.941 | 1.000 | 1044 | tags=94%, list=74%, signal=357% |
| 340 | EWSR1\_TARGET\_GENES |  | 82 | -0.10 | -0.71 | 0.847 | 0.939 | 1.000 | 229 | tags=20%, list=16%, signal=22% |
| 341 | ZNF680\_TARGET\_GENES |  | 73 | -0.10 | -0.69 | 0.893 | 0.952 | 1.000 | 161 | tags=15%, list=11%, signal=16% |
| 342 | MGGAAGTG\_GABP\_B |  | 73 | -0.10 | -0.69 | 0.870 | 0.952 | 1.000 | 84 | tags=10%, list=6%, signal=10% |
| 343 | TAL1BETAITF2\_01 |  | 18 | -0.16 | -0.69 | 0.821 | 0.950 | 1.000 | 195 | tags=22%, list=14%, signal=25% |
| 344 | TOP2B\_TARGET\_GENES |  | 61 | -0.10 | -0.69 | 0.882 | 0.948 | 1.000 | 393 | tags=36%, list=28%, signal=48% |
| 345 | ZNF768\_TARGET\_GENES |  | 87 | -0.09 | -0.69 | 0.924 | 0.950 | 1.000 | 535 | tags=48%, list=38%, signal=73% |
| 346 | ZNF563\_TARGET\_GENES |  | 51 | -0.11 | -0.68 | 0.891 | 0.954 | 1.000 | 1264 | tags=100%, list=90%, signal=925% |
| 347 | GATA3\_01 |  | 17 | -0.16 | -0.67 | 0.879 | 0.956 | 1.000 | 944 | tags=88%, list=67%, signal=263% |
| 348 | FOXG1\_TARGET\_GENES |  | 62 | -0.10 | -0.67 | 0.901 | 0.959 | 1.000 | 1178 | tags=95%, list=83%, signal=551% |
| 349 | GCTNWTTGK\_UNKNOWN |  | 19 | -0.15 | -0.67 | 0.877 | 0.959 | 1.000 | 298 | tags=32%, list=21%, signal=39% |
| 350 | ZBTB10\_TARGET\_GENES |  | 191 | -0.07 | -0.66 | 0.975 | 0.961 | 1.000 | 590 | tags=51%, list=42%, signal=76% |
| 351 | BCL6B\_TARGET\_GENES |  | 24 | -0.13 | -0.66 | 0.876 | 0.959 | 1.000 | 83 | tags=13%, list=6%, signal=13% |
| 352 | MAX\_01 |  | 30 | -0.12 | -0.66 | 0.898 | 0.960 | 1.000 | 20 | tags=7%, list=1%, signal=7% |
| 353 | CREB\_Q4 |  | 22 | -0.14 | -0.65 | 0.872 | 0.963 | 1.000 | 484 | tags=50%, list=34%, signal=75% |
| 354 | RFX1\_01 |  | 23 | -0.13 | -0.64 | 0.907 | 0.965 | 1.000 | 394 | tags=39%, list=28%, signal=53% |
| 355 | ZNF577\_TARGET\_GENES |  | 45 | -0.10 | -0.64 | 0.896 | 0.964 | 1.000 | 189 | tags=16%, list=13%, signal=17% |
| 356 | ZNF766\_TARGET\_GENES |  | 118 | -0.08 | -0.64 | 0.947 | 0.963 | 1.000 | 115 | tags=9%, list=8%, signal=9% |
| 357 | INSM2\_TARGET\_GENES |  | 71 | -0.09 | -0.63 | 0.942 | 0.966 | 1.000 | 236 | tags=18%, list=17%, signal=21% |
| 358 | CASP8AP2\_TARGET\_GENES |  | 22 | -0.13 | -0.63 | 0.917 | 0.967 | 1.000 | 1008 | tags=91%, list=71%, signal=313% |
| 359 | ZNF318\_TARGET\_GENES |  | 108 | -0.08 | -0.62 | 0.975 | 0.968 | 1.000 | 537 | tags=46%, list=38%, signal=69% |
| 360 | YNGTTNNNATT\_UNKNOWN |  | 34 | -0.11 | -0.62 | 0.938 | 0.968 | 1.000 | 455 | tags=41%, list=32%, signal=59% |
| 361 | ARNT\_02 |  | 28 | -0.12 | -0.61 | 0.923 | 0.973 | 1.000 | 1247 | tags=100%, list=88%, signal=843% |
| 362 | HOXC6\_TARGET\_GENES |  | 111 | -0.08 | -0.61 | 0.961 | 0.971 | 1.000 | 366 | tags=29%, list=26%, signal=36% |
| 363 | TERF1\_TARGET\_GENES |  | 31 | -0.11 | -0.61 | 0.914 | 0.969 | 1.000 | 253 | tags=23%, list=18%, signal=27% |
| 364 | WCAANNNYCAG\_UNKNOWN |  | 29 | -0.11 | -0.59 | 0.945 | 0.978 | 1.000 | 1258 | tags=100%, list=89%, signal=903% |
| 365 | RGAGGAARY\_PU1\_Q6 |  | 33 | -0.11 | -0.59 | 0.949 | 0.977 | 1.000 | 403 | tags=36%, list=29%, signal=50% |
| 366 | IRF1\_01 |  | 21 | -0.12 | -0.58 | 0.932 | 0.979 | 1.000 | 340 | tags=33%, list=24%, signal=43% |
| 367 | IRF9\_TARGET\_GENES |  | 43 | -0.09 | -0.57 | 0.942 | 0.977 | 1.000 | 315 | tags=26%, list=22%, signal=32% |
| 368 | KLF14\_TARGET\_GENES |  | 20 | -0.12 | -0.57 | 0.956 | 0.978 | 1.000 | 83 | tags=10%, list=6%, signal=10% |
| 369 | MYCMAX\_B |  | 20 | -0.12 | -0.55 | 0.967 | 0.982 | 1.000 | 1015 | tags=90%, list=72%, signal=316% |
| 370 | LMTK3\_TARGET\_GENES |  | 45 | -0.09 | -0.54 | 0.950 | 0.983 | 1.000 | 115 | tags=11%, list=8%, signal=12% |
| 371 | ZNF618\_TARGET\_GENES |  | 37 | -0.10 | -0.54 | 0.965 | 0.981 | 1.000 | 441 | tags=38%, list=31%, signal=54% |
| 372 | HDGF\_TARGET\_GENES |  | 67 | -0.08 | -0.53 | 0.975 | 0.982 | 1.000 | 204 | tags=16%, list=14%, signal=18% |
| 373 | ZNF513\_TARGET\_GENES |  | 72 | -0.08 | -0.52 | 0.978 | 0.984 | 1.000 | 321 | tags=25%, list=23%, signal=31% |
| 374 | ZNF250\_TARGET\_GENES |  | 50 | -0.07 | -0.43 | 0.991 | 0.997 | 1.000 | 347 | tags=26%, list=25%, signal=33% |
Table: Gene sets enriched in phenotype **NCAPD3\_MUT (3 samples) **[plain text format]****
